# The Effect of Allergic Conjunctivitis on Refractive Error in the Pediatric Population

**DOI:** 10.7759/cureus.84417

**Published:** 2025-05-19

**Authors:** Charles Zhang, Sinan Ersan, Treefa Shwani, Andrew Beiter, Yousef Yousef, Margaret M DeAngelis, Andrew L Reynolds

**Affiliations:** 1 Department of Ophthalmology, Ross Eye Institute, Buffalo, USA; 2 Department of Ophthalmology, University at Buffalo Jacobs School of Medicine and Biomedical Sciences, Buffalo, USA; 3 Neuroscience Graduate Program, University at Buffalo Jacobs School of Medicine and Biomedical Sciences, Buffalo, USA; 4 Department of Ophthalmology, West Virginia University Eye Institute, Morgantown, USA; 5 Genetics, Genomics and Bioinformatics Graduate Program, University at Buffalo Jacobs School of Medicine and Biomedical Sciences, Buffalo, USA; 6 Department of Ophthalmology and Visual Sciences, University of Utah School of Medicine, Buffalo, USA; 7 Department of Population Health Sciences, University of Utah School of Medicine, Buffalo, USA; 8 Department of Ophthalmology, Veterans Administration Western New York Healthcare System, Buffalo, USA; 9 Department of Biochemistry, University at Buffalo Jacobs School of Medicine and Biomedical Sciences, Buffalo, USA

**Keywords:** allergic conjunctivitis, astigmatism, cylindrical refractive error, ocular allergies, refractive error

## Abstract

Introduction

Refractive error is one of the most common causes of visual impairment in the world, negatively affecting a patient’s quality of life, economic opportunities and more. Although numerous studies have explored the impact of severe ocular allergies on refractive error, there is limited research on how relatively mild forms of ocular allergies may affect vision. This study aims to assess the impact of allergic conjunctivitis, a milder and more common ocular allergy, on the development and progression of refractive error in the pediatric population.

Methods

This was a retrospective study that reviewed the records (2015-2018) of 198 established pediatric patients from the Ross Eye Institute: 90 with a new diagnosis of allergic conjunctivitis, and 108 age- and sex-matched controls. All refractions were performed by a single board-certified and fellowship-trained pediatric ophthalmologist utilizing retinoscopy, the gold standard for measuring refractive error in the pediatric population. T-tests were used to analyze continuous variables, and chi-square tests were used for categorical variables. Linear regression was used to identify factors associated with refractive error.

Results

The average age of the patient population was 9.7 years, with 42% males. Children with allergic conjunctivitis were found to have a significantly higher incidence of cylindrical refractive error compared to controls (56% vs 34%, p < 0.001), with increased cylindrical power (1.20 ± 0.07 D vs 0.80 ± 0.08 D; p < 0.001), but no difference in spherical equivalent (-0.83 ± 0.27 vs -0.66 ± 0.21, p = 0.64). Additionally, compared to the refraction obtained one year prior to the diagnosis of allergic conjunctivitis, patients were found to have a greater increase in cylindrical power compared to controls (0.13 ± 0.04 D vs 0.01 ± 0.02 D, p = 0.007), with no significant difference in the type of cylindrical refractive error.

Conclusions

Our study demonstrates that allergic conjunctivitis may prognosticate the development and progression of cylindrical refractive error in children.

## Introduction

Refractive error is one of the most common causes of visual impairment and blindness, affecting over 2.2 billion people [1, 2]. Uncorrected refractive error negatively affects education, quality of life, and economic opportunities, ultimately costing the global economy over $410 billion each year [1-3]. Given the detrimental impact of this condition, refractive error has been gaining increasing interest from national and global organizations over the last decade. In 2019, the World Health Organization released the *World Report on Vision*, highlighting the necessity for action aimed at addressing refractive error across the world [4]. Studies have since shown that the rate of refractive error has been rising, with over 160 million people estimated to be blind or living with moderate-to-severe visual impairment due to the inability to obtain refractive correction [2]. In 2024, the National Academies of Sciences, Engineering, and Medicine (NASEM) released a landmark report underscoring the vast scope and impact of refractive error [5]. In response, the American Academy of Optometry petitioned for refractive error to be reclassified as a disease, emphasizing the need to fully recognize the widespread effects of this condition.

Refractive error is defined as the inability to accurately focus light onto the retina, the layer of cells responsible for vision in the eye [6]. Consequently, refractive error typically arises and progresses during childhood, when the eye is developing and changing in shape [6]. The gold standard for identifying refractive error in this age group is retinoscopy, an objective technique that determines the magnitude and type of refractive error by observing the reflection of light from the retina [7]. Unlike other methods of diagnosing refractive error, including vision screeners and subjective refraction techniques, retinoscopy does not rely on patient responses and is therefore agnostic in nature, especially in children who cannot express their thoughts [7]. Although identification and treatment of refractive error is crucial in mitigating the detrimental effects of this condition, recognizing modifiable risk factors is equally important. Currently, family history/genetics of refractive error, severe ocular allergies, trauma, and prior ocular surgeries have been shown to increase rates of refractive error in children [6]. However, the effects of common ocular diseases and their association with refractive error in childhood are not well understood. 

Ocular allergies, one of the most common ocular diseases, affect 15-20% of the United States population [8]. Symptoms are highly variable but typically include redness, swollen eyelids, watering, itching, pain, and photophobia [9]. Severe forms of ocular allergies, including vernal conjunctivitis, have been associated with increased refractive error, potentially resulting from corneal topographical changes caused by repeated eye rubbing [10-12]. However, while there are a myriad of studies evaluating the effect of severe ocular allergies on refractive error, this same association has not been established with the most common form of ocular allergy in the pediatric population, allergic conjunctivitis (AC). Given the severe consequences associated with an increase in refractive error, it is crucial to identify if an association exists between AC, a potentially modifiable condition, and refractive error.

## Materials and methods

This is a retrospective record review conducted at the Ross Eye Institute, The State University of New York (SUNY) at Buffalo. The study was approved by the SUNY University at Buffalo Institutional Review Board, and the research adheres to the tenets of the Declaration of Helsinki.

Records of children under the age of 18 who visited the Ross Eye Institute between October 2015 and September 2018 were reviewed. The inclusion criterion for the study population was children with a new diagnosis of AC. The exclusion criteria included the presence of any other ocular diagnoses, chronic systemic diagnoses, chronic systemic medications, or use of antihistamine eye drops. A total of 90 patients with AC were reviewed. Records of 108 patients with a diagnosis of esophoria, a benign alignment disorder, were chosen as the control group. The same exclusion criteria were applied to this group in order to reduce the risk of confounding variables. Esophoria is often used by pediatric ophthalmologists as a reimbursement-driven code for patients who are not diagnosed with any other ocular conditions, and does not impact refractive error [13]. Both eyes of each patient were included in this study. The patient data was extracted from MedFlow Version 8.2 Electronic Medical Record (MedFlow, Inc., San Francisco, USA). Patients’ age, sex, race, and refractive error of each eye were collected.

At the time of diagnosis, all patients with allergic conjunctivitis had at least one symptom of the condition, including, but not limited to, itching, redness, blurred vision, and photophobia [8]. The diagnosis of allergic conjunctivitis was formally given to all patients after a slit lamp examination was performed by a fellowship-trained and board-certified pediatric ophthalmologist. Patients were prescribed olopatadine, a combination antihistamine and mast-cell stabilizer, at this visit to be taken once daily or twice daily, depending on the formulation approved by their insurance. 

**Figure 1:**
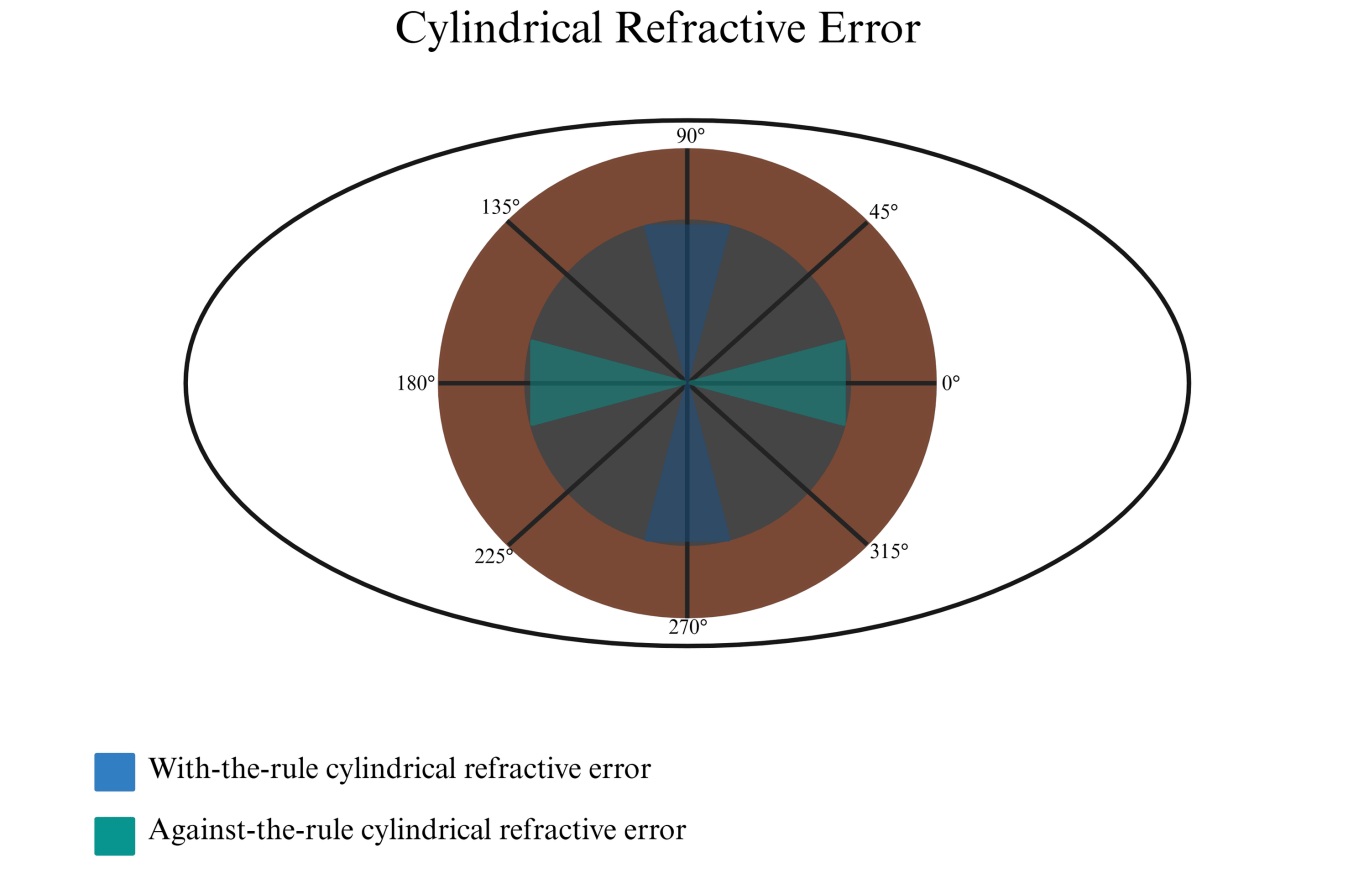
Types of Cylindrical Refractive Error Figure 1 is a visual illustration of the types of cylindrical refractive error, including with-the-rule when the steeper axis is between 75-105° or 255-285°, against-the-rule when the steeper axis is between 165-195° or 345-15°, and oblique for all other degrees as previously described [19]. Image Credits: Sinan Ersan.

In order to determine refractive changes in untreated patients with allergic conjunctivitis, the refractive error of patients from a prior visit (≥ one year prior) was obtained. All refractions, for both cases and controls, were obtained by a single fellowship-trained and board-certified pediatric ophthalmologist through retinoscopy, the gold standard for measuring refractive error in pediatric patients [7]. Each patient’s refractive error was documented using the general format: spherical power, cylindrical power, and axis. Spherical equivalent (SE) was calculated using the formula *SE = spherical power + 1/2(cylindrical power)*, as previously described in the literature [14, 15]. When classifying refractive error, myopia (near-sightedness) was considered a spherical equivalent of ≤ -0.5 diopters (D), hyperopia (far-sightedness) was considered a spherical equivalent of ≥ 2 D, and emmetropia was any value between -0.5 D and 2.0 D [16, 17]. When classifying cylindrical power, any value ≥ 1 D was considered to be cylindrical refractive error [18]. Patients with cylindrical refractive error were further classified as having with-the-rule (WTR), against-the-rule (ATR), or oblique, as illustrated in Figure 1. This classification of cylindrical refractive error has been previously described in the literature [19]. BioRender (BioRender, Toronto, Canada) was accessed on January 17th, 2025, and used to create Figure 1.

Data analysis

Data analysis was performed using R Studio version 1.1.456 (R Foundation for Statistical Computing, Vienna, Austria) with an α of 0.05. A power analysis was performed to determine sample size. A sample size of 63 was proposed by doing a power calculation that gave a power of 0.80 with a proposed effect size of d = 0.5. Independent t-tests were applied for continuous variables and chi-square tests for categorical variables to compare baseline characteristics of cases and controls. Univariate linear regression was performed to identify variables associated with refractive error. Following this, bidirectional stepwise multivariate linear regression was performed on all univariate coefficients with a p-value < 0.10. P-values <0.05 were considered statistically significant.

## Results

Index visit

**Figure 2:**
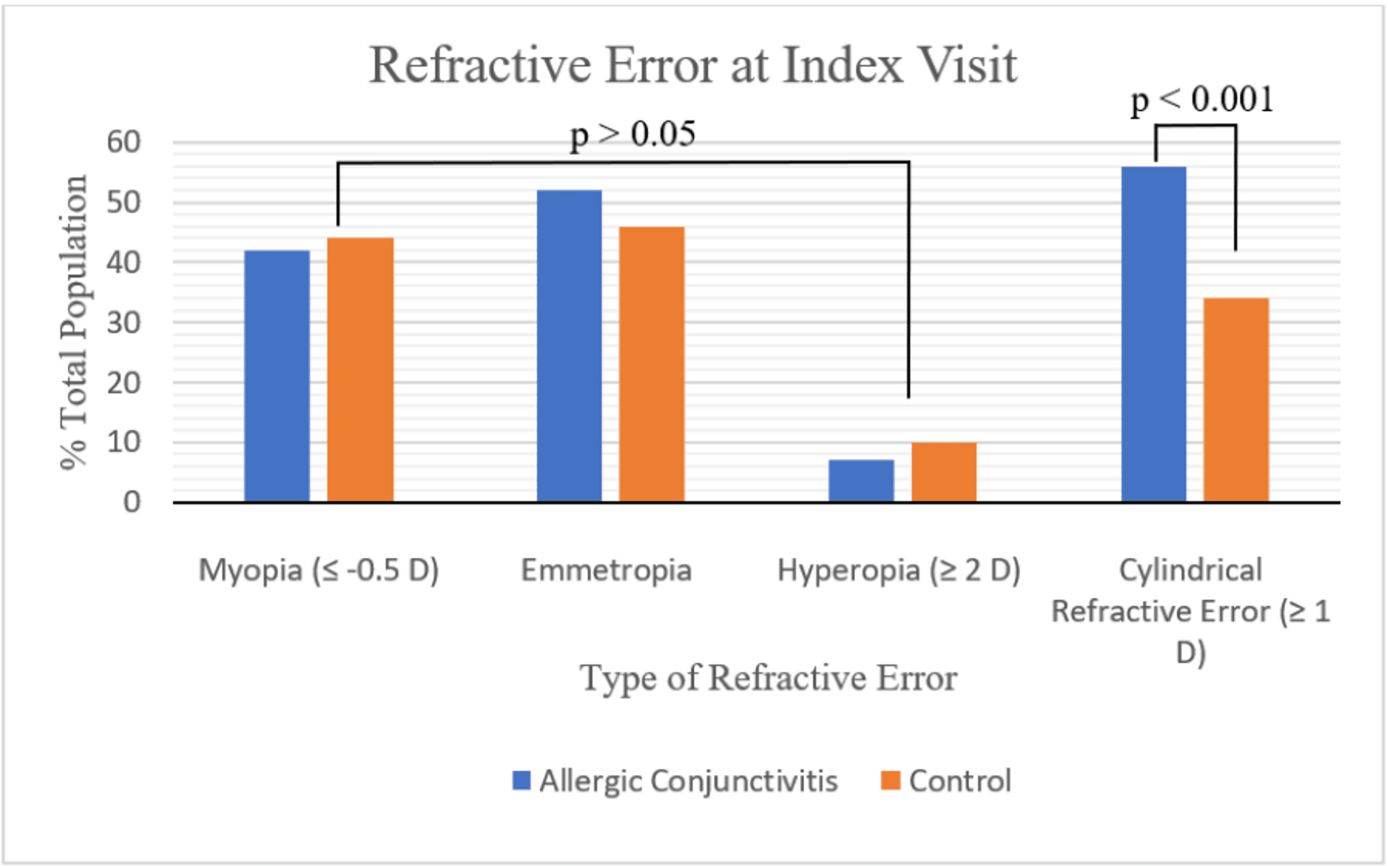
Types of Refractive Error at Index Visit Figure 2 depicts the types of refractive error seen between children with allergic conjunctivitis and controls at the day of diagnosis (index visit). Chi-square tests were performed to compare the two groups.

**Figure 3:**
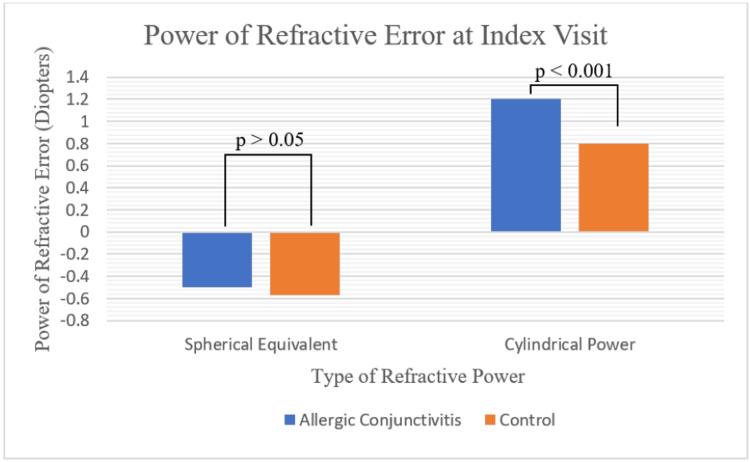
Spherical Equivalent and Cylindrical Power at Index Visit Figure 3 compares the spherical equivalent and cylindrical power in children with allergic conjunctivitis and controls at the day of diagnosis (index visit). Independent t-tests were performed to compare the two groups.

A total of 90 patients with AC and 108 controls were reviewed. No significant difference in baseline characteristics was found (Table 1). The refraction performed on the day of diagnosis of AC (index visit) was obtained through retinoscopy. As depicted in Figure 2 and Figure 3, there was a significantly higher proportion of patients with cylindrical refractive error in the conjunctivitis group (56% vs 34%), which also corresponded to a higher average cylindrical power (1.20 ± 0.07 D vs 0.80 ± 0.08 D). No significant difference in spherical equivalent was found between groups (-0.50 ± 0.18 D vs -0.57 ± 0.16 D). Similarly, when categorizing spherical equivalent into myope (42% vs 44%), emmetrope (52% vs 46%), and hyperope (7% vs 10%), no significant differences were seen. When assessing the type of cylindrical refractive error, no difference between the percentage of WTR (82% vs 84%), ATR (6% vs 5%), and oblique (12% vs 11%) was found (p=0.97).

**Table 1:** Baseline Characteristics of Patients at Index Visit Table 1 describes the baseline characteristics of patients diagnosed with allergic conjunctivitis and controls. Independent t-tests were applied for continuous variables and chi-square tests for categorical variables. Information regarding race was only available for 33 (37%) patients with allergic conjunctivitis and 56 (52%) controls.

Characteristics	Allergic Conjunctivitis (N = 90)	Controls (N = 108)	T-value/ Chi-square value	P-value
Age (Years)	9.3 ± 0.5	10.0 ± 0.4	T-value = 0.994	0.32
Sex (Male)	37 (41%)	46(42%)	Chi-square value = 0.187	0.83
Race	20 (61%) White	36 (64%) White	Chi-square value = 0.385	0.92
	7 (21%) African American/ Black	10 (18%) African American/ Black		
	6 (18%) Other	10 (18%) Other		

Linear regression was performed to determine which variables were associated with higher cylindrical power. Univariate regression showed that the diagnosis of AC (1.51, 95% confidence internal (CI) 1.40-1.63 (p<0.001)) and degree of myopia (1.09 per D, 95% CI 1.07-1.11 (p<0.001)) were significant predictors of increased cylindrical power. Demographic variables were not significant in our population. In multivariate analysis, only AC was associated with higher cylindrical power (1.49, 95% CI 1.39-1.60 (p<0.001)).

To evaluate the seasonality of the disease, we assessed the seasons by which patients presented for their index visit evaluation. A total of 30, 33, 19, and 22 patients with AC and 32, 34, 16, and 26 controls presented in winter, spring, summer, and fall, respectively (p=0.89). The refractive error and cylindrical power were not significantly associated with the season of presentation (p=0.85 and p=0.70, respectively).

Refraction prior to the index visit

Refraction was obtained prior to the index visit in 38 (42%) patients who would later be diagnosed with AC and 69 (64%) controls (Table 2). The average days prior to the index visit were 499±40 and 498±20, respectively (p=0.98). At this time point, patients who would later be diagnosed with allergic conjunctivitis had a higher rate of cylindrical refractive error, as well as a greater average cylindrical power. Similar to the index visit refraction, no significant difference in average spherical equivalent, myopia, emmetropia, or hyperopia was found. Additionally, there was no significant difference in the type of cylindrical refractive error (88% vs 80%, 5% vs 4%, and 7% vs 16% for WTR, ATR, and oblique, respectively (p=0.45)).

**Table 2:** Refractive Characteristics of Patients Prior to the Index Visit Table 2 describes the types of refractive error seen between children with allergic conjunctivitis and controls at the visit prior to diagnosis. Independent t-tests were applied for continuous variables and chi-square tests for categorical variables.

	Allergic Conjunctivitis (N=38)	Controls (N=69)	T-value/ Chi-square value	P-value
Myopia	21 (55%)	37 (54%)	Chi-square value = 0.103	0.95
Emmetropia	14 (37%)	26 (38%)		
Hyperopia	3 (8%)	6 (9%)		
Cylindrical Refractive Error	21 (55%)	25 (36%)	Chi-square value = 6.3	0.012
Cylindrical Power (Diopters)	1.29 ± 0.14	0.91 ± 0.10	T-value = 2.20	0.03
Spherical Equivalent (Diopters)	-0.83 ± 0.27	-0.66 ± 0.21	T-value = 0.41	0.64

Comparison between the visits

When compared to the index visit, patients with AC had a significantly greater increase in cylindrical power between visits (0.13 ± 0.04 D vs 0.01 ± 0.02 D (p = 0.007)). At the level of the individual, a statistically significant percentage of children with AC (33% AC vs 13% controls (p=0.002)) had an increase in cylindrical power greater than 0.5 D between the two visits. When linear regression was performed to evaluate the association between increased cylindrical power in this time interval, the diagnosis of conjunctivitis (1.13 ± 0.04 (p=0.006)), but not the degree of myopia (1.02 ± 0.01 (p=0.06)), was associated with increasing cylindrical power. Other demographic variables were not significantly associated with the progression of cylindrical refractive error.

## Discussion

Utilizing a rigorous methodological approach, in an unbiased manner, we report the following: allergic conjunctivitis (AC) in the pediatric population is associated with an increased incidence and rate of progression of cylindrical refractive error, without a significant difference in the type of cylindrical refractive error. One prior study, conducted in South Korea, demonstrated a similar relationship between allergic conjunctivitis and cylindrical power [20], however, there are several methodological differences that distinguish our study. First, while the South Korean study examined refractive error at a single time point, the current study builds upon this by exploring longitudinal changes in patients with undiagnosed AC. Second, our study utilizes retinoscopy, the gold standard for measuring refractive error in children, which is different than the Spot Vision Screener utilized by the study from South Korea. Spot Vision Screeners have a reported sensitivity of 66.7% and rely heavily on patient cooperation, limiting their reliability for accurately measuring refractive error in children [21]. Third, the current study included a diverse population of children (63% White and 19% African American and/or Black). The study from South Korea did not report race in their results; however, the patients were recruited from local elementary schools, possibly indicating a homogenous population [20].

In the current study, we demonstrate a statistically significant trend where the children who would later be diagnosed with AC display a more pronounced increase in both the rate of cylindrical refractive error and magnitude of cylindrical power over time. As AC is a chronic condition, it's likely that, at the time of the earlier visit, these children were already beginning to manifest findings of AC. [22]. However, given the seasonal characteristic or potential subacute presentation of AC during the visit, the early symptoms might have eluded detection by both the ophthalmologist and the patient’s guardians. This suggests that the underlying development of AC could be subtly influencing the progression of cylindrical refractive error even before its formal diagnosis, further strengthening the association between these conditions.

Prior studies have focused on the relationship between severe ocular diseases, such as vernal keratoconjunctivitis, and refractive changes [11, 12]. These studies report corneal topographical changes as high as 71%, significantly higher than those observed in our study, with patients presenting with higher cylindrical power [11]. Further, there was an association noted with keratoconus, a serious corneal ectatic disease where the cornea gradually deforms and assumes an irregular, cone-like shape [11, 12]. Additional studies aimed at identifying the underlying mechanisms through which vernal keratoconjunctivitis leads to cylindrical refractive error have implicated mechanical contact, particularly eye rubbing, as a partial contributor to observed refractive changes [23]. AC, while relatively less severe in symptoms compared to the severe forms of ocular allergies, is also associated with frequent eye rubbing, particularly among children [24].

While our study identified a statistically significant relationship between AC and cylindrical refractive error, interpreting its clinical significance is challenging. The average cylindrical power found in our study was 1.29 D, which, despite being statistically significant, may not be enough on its own to induce amblyopia, a visual development disorder typically resulting from anisometropia (a difference in refractive error between the two eyes). Amblyopia is more commonly associated with an anisometropic cylindrical refractive error of >2.0 D or an isometropic cylindrical refractive error of >2-3 D [25, 26]. The threshold for amblyopia may be lower in cases of oblique cylindrical refractive error, but our data demonstrated that the type of cylindrical refractive error was not altered by AC, which predominantly remains “with-the-rule”. However, it's crucial to note that our study demonstrated a progressive increase in cylindrical refractive error over time in children who would develop AC. This suggests that, if left untreated, the condition may potentially worsen, resulting in children with higher baseline cylindrical power who may develop amblyopia. This is particularly significant because amblyopia represents the leading cause of preventable vision loss in developed countries [26].

This study has some limitations. Firstly, our diagnosis relies on the presence of typical slit-lamp findings associated with AC, including papillae, hyperemia, and chemosis, as well as patient-reported symptoms. Patients may only experience symptoms seasonally, which may underrepresent the true population suffering from AC [22]. Although we did not find any significance between seasonality and refractive error, factors such as availability to schedule an appointment and severity of symptoms may influence office visit dates. Secondly, the severity of AC was not graded in a uniform manner, and thus, could not be examined in this study. There are several reports that have proposed grading systems; however, there is currently no universally accepted categorization of AC severity that incorporates all aspects of the clinical exam [27, 28].

## Conclusions

In conclusion, this study highlights a significant association between AC and the development and progression of cylindrical refractive error in children. Our findings indicate that AC may serve as a risk factor for cylindrical refractive error, potentially increasing the likelihood of amblyopia and the need for corrective eyewear. Future research should investigate whether effective treatment of allergic conjunctivitis can mitigate these effects and slow the progression of refractive error in the pediatric population.
